# Changes in Response Inhibition, Visual Anticipation and Verbal Fluency During Vagus Nerve Stimulation Therapy in Patients With Drug‐Resistant Epilepsy

**DOI:** 10.1002/brb3.70176

**Published:** 2024-12-06

**Authors:** Niina Lähde, Pabitra Basnyat, Jani Raitanen, Leena Kämppi, Kai Lehtimäki, Eija Rosti‐Otajärvi, Jukka Peltola

**Affiliations:** ^1^ Department of Neurology Tampere University Hospital Tampere Finland; ^2^ Faculty of Medicine and Health Technology Tampere University Tampere Finland; ^3^ Faculty of Social Sciences, Health Sciences Tampere University Tampere Finland; ^4^ UKK Institute for Health Promotion Research Tampere Finland; ^5^ Epilepsia Helsinki, Member of EpiCARE ERN, Department of Neurology Helsinki University Hospital and University of Helsinki Helsinki Finland; ^6^ Department of Neurosurgery Tampere University Hospital Tampere Finland; ^7^ Department of Rehabilitation and Psychosocial Support Tampere University Hospital Tampere Finland

**Keywords:** attention and executive functions, cognition, drug‐resistant epilepsy, vagus nerve stimulation

## Abstract

**Background:**

The effect of vagus nerve stimulation (VNS) on cognitive domain of attention and executive functions (AEFs) has not been extensively researched. This study was set up to investigate performance variability on cognitive tests assessing AEFs in drug‐resistant epilepsy (DRE) patients receiving VNS therapy during a follow‐up of up to 5 years.

**Methods:**

Thirty‐three DRE patients were assessed with the interference, maze, and written verbal fluency tests as a part of EpiTrack screening before and after VNS implantation through repeated follow‐ups according to the clinical VNS protocol. A linear mixed‐effects model was used to analyse changes in test scores.

**Results:**

Maze performance improved significantly by an average of 0.20 s per month (95% confidence interval (CI): –0.365 to –0.041; *p* = 0.014). Interference performance improved by an average of 0.05 s per month (*p* = 0.207) and number of words increased by an average of 0.03 words per month (*p* = 0.079) on the verbal fluency test. On the maze test, patients with psychiatric comorbidities improved the most (0.52 s/month, *p* = 0.001), while on the interference test, patients with frontal lobe epilepsy (FLE), those taking 1–2 antiseizure medications (ASMs) and patients with focal to bilateral tonic–clonic seizures improved the most (0.14 s/month, *p* = 0.005; 0.14 s/month, *p* = 0.033 and 0.16 s/month, *p* = 0.087, respectively). For verbal fluency, no clinically meaningful improvement was noted in any of the groups.

**Conclusion:**

During the follow‐up, maze performance markedly improved, while performance on the interference and verbal fluency tasks remained relatively stable at the group level. Accordingly, visual anticipation and planning improved during VNS therapy whereas response inhibition was unchanged at the group level despite significant enhancements in patients with FLE and those taking 1–2 ASM. Furthermore, the presence of psychiatric comorbidities correlated with even greater improvement on maze performance.

## Graphical abstract

33 patients with drug‐resistant epilepsy were treated with VNS and followed‐up up to 5 years. Patients were evaluated with the maze, interference and written verbal fluency tests as a part of EpiTrack screening before and after VNS implantation through repeated follow‐ups. The results showed that visual anticipation and planning improved significantly on a group level during follow‐up, with marked enhancements observed in DRE patients with psychiatric comorbidities during VNS therapy. Patients with frontal lobe epilepsy and those taking fewer than three ASMs improved significantly in response inhibition.

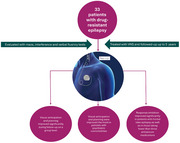

**Abbreviations**
AEFs: attention and executive functions
ASM: antiseizure medication
DRE: drug‐resistant epilepsy
EFs: executive functions
FAS: focal aware seizure
FBTCS: focal to bilateral tonic‒clonic seizure
FIAS: focal impaired awareness seizure
FLE: frontal lobe epilepsy
LME: linear mixed‐effects
TLE: temporal lobe epilepsy
VNS: vagus nerve stimulation

## 1 Introduction

Cognitive impairments are present in 75% of patients with drug‐resistant epilepsy (DRE) leading to a significant impact on patients' daily functioning (Lähde et al. 2021; Keezer, Sisodiya, and Sander 2016). The efficacy of vagus nerve stimulation (VNS) has been well‐documented for seizure control in epilepsy (Ben‐Menachem et al. 1994; Elliott et al. 2011). A recent meta‐analysis examining the impact of VNS on cognition in DRE patients revealed no significant changes in either attention or executive functions (EFs) following VNS therapy. However, the analysis underscored a paucity of high‐quality data (Kong et al. 2024). Nevertheless, some evidence suggests that VNS may induce favourable short‐term improvements in working memory and attention (Sun et al. 2017; Aniwattanapong et al. 2022).

**Plain Language Summary**

Not much research has been done on the effect of vagus nerve stimulation therapy on attention and executive functions, although problems in these cognitive abilities are common in patients with drug‐resistant epilepsy. In this study, we investigated how vagus nerve stimulation therapy affects cognitive abilities in patients with drug‐resistant epilepsy, focusing on attention and executive functions. We included 33 patients who were followed‐up up to 5 years and evaluated with three different cognitive tests before starting vagus nerve stimulation therapy and repeatedly during the treatment period. Results showed notable improvements in planning skills over time, particularly in patients suffered from psychiatric comorbidities. However, improvement in verbal fluency and inhibitory control abilities was not observed during the follow‐up period.

Executive functions are a collection of complex cognitive abilities that are essential for adaptive and goal‐oriented behaviour. Attention, which is the ability to select information to be processed with priority, is critical for high‐level cognition (Diamond 2013). In a previous study, we observed a gradual and clinically meaningful improvement in attention and executive functions (AEFs) performance measured by the EpiTrack total score among DRE patients following VNS therapy (Lähde et al. 2024b). EpiTrack is a screening tool for the assessment of AEFs in epilepsy patients (Lutz and Helmstaedter 2005). Furthermore, in another study involving an extended group of DRE patients receiving VNS therapy, we evaluated performance variability on Trail‐Making Test Parts A and B and the Digit Span Backward Task and identified the most substantial enhancement on Trail‐Making Test Part B (Lähde et al. 2024a), which specifically evaluates set‐shifting. The Trail‐Making Test and Digit Span Backward Task are included in the EpiTrack evaluation along with an interference test, a maze test and a written phonemic verbal fluency test (Helmstaedter 2012).

The EpiTrack interference test primarily assesses response inhibition (Lehrl and Fischer 1997), which refers to the ability of individuals to resist a predominant, automatic or learned behaviour that might be inappropriate or irrelevant in the present context (Diamond 2013). The maze task specifically evaluates planning and visual anticipation. Verbal fluency tests typically have two elements—phonemic fluency and semantic fluency (Lezak et al. 2004). These tasks involve both verbal ability and executive control, with the phonemic fluency task imposing more demands on EFs (Shao et al. 2014).

The present study evaluated potential performance variability on these three aforementioned cognitive tests assessing primarily response inhibition, visual anticipation or verbal fluency in a cohort of DRE patients receiving VNS therapy during a follow‐up of up to 5 years.

## 2 Materials and Methods

### 2.1 Study Design

This was a noninterventional study in which data were collected prospectively but analysed retrospectively from a VNS quality registry at Tampere University Hospital. Due to the registry‐based nature of the data, ethics committee approval was not required according to the Finnish Law on Research. Access to the VNS quality register was granted by the Tampere University Hospital Research, Development and Innovation Centre.

### 2.2 Patients and Follow‐Up

This study included 33 DRE patients who were implanted with VNS (Model 106 (Aspire) or Model 1000 (SenTiva) at Tampere University Hospital and were evaluated with the maze, interference, and written verbal fluency tests prior to implantation, at 6 and12 months after implantation, and yearly thereafter as a part of standard clinical VNS protocol. For this study, all patients implanted with VNS from September 2, 2015, to February 25, 2021, with a minimum follow‐up of 12 months until the end of February 2022 and at least two postimplantation assessments were included. These patients are described in more detail in a previous publication (Lähde et al. 2024b).

Furthermore, due to the COVID‐19 pandemic, scheduled appointments did not always occur according to our protocol. Therefore, changes in the test scores over time were analysed using a linear mixed‐effects (LME) model to compensate for the variation in follow‐up duration when predicting changes in the test scores over 5 years. The actual timing of the assessments is presented in Figure S1.

### 2.3 Patient Characteristics

We retrospectively extracted information on age at baseline, sex, concomitant psychiatric comorbidities (either current or in the past), age at epilepsy onset, epilepsy duration, aetiology and type of epilepsy, predominant seizure type and frequency during the 12 months prior to VNS implantation and 3 months prior to each postimplantation assessment, current antiseizure medication (ASM) use, and model and duration of VNS from the VNS quality registry.

Epilepsy type was categorized as temporal lobe epilepsy (TLE), frontal lobe epilepsy (FLE), generalized epilepsy or other (one case of parietal lobe epilepsy; three cases of multilobar epilepsy; and two cases of multifocal epilepsy). Seizure type was classified by video‐electroencephalogram findings and seizure semiology. The predominant seizure type (focal aware seizure (FAS), focal impaired awareness seizure (FIAS), and focal to bilateral tonic‒clonic seizure (FBTCS)) for each patient was defined as the most disabling seizure type noted in the medical records as determined by the physician (Orosz et al. 2014). Patients with FAS and FIAS were combined into a single seizure type group in the analysis. One patient was seizure‐free at baseline (predominant seizure type FBTCS), and the frequency of the predominant seizure type was not available for one patient (FIAS). These two patients were excluded from the analysis on the effect of predominant seizure type on test performance.

All patients were treated with ASMs (range 1 to 4) in addition to VNS. We defined ASM burden reduction as ASM withdrawal and/or dose reduction and ASM burden increase as ASM addition and/or dose increase during the follow‐up. Baseline AEF performance was determined by the EpiTrack total score, where a score of 32 points signifies normal performance, scores between 29 and 31 indicate mild impairment, and scores of 28 or lower represent severe impairment. The clinical characteristics of the patients are presented in Table 1.

**TABLE 1:** Demographics and clinical characteristics of the patients.

Total patients (*n* = 33)	Descriptives
Age at baseline in years (median, (IQR))	32 (27–41)
Sex (female/male)	19/14
Psychiatric comorbidity	
*Yes (n, %)*	9 (27.3)
*Present/past*	5/4
*No (n, %)*	24 (72.7)
Age at epilepsy onset in years (median, (IQR))	15 (9.5–20)
Epilepsy duration in years (median, (IQR))	17 (10–24.5)
Baseline AEF performance	
*Normal*	12 (36.4)
*Mildly impaired*	8 (24.2)
*Severely impaired*	13 (39.4)
ILAE etiology (*n*, %)	
Structural	10 (30.3)
*Cortical dysplasia*	2 (6.1)
*Vascular lesion*	3 (9.1)
*Cavernoma*	1 (3.0)
*Av‐malformation*	1 (3.0)
*Brain trauma*	1 (3.0)
*Late effects of radiation*	1 (3.0)
*Hippocampal sclerosis*	1 (3.0)
*Immune*	4 (12.1)
*Autoimmune encephalitis*	4 (12.1)
Genetic	1 (3.0)
Unknown	18 (54.5)
Epilepsy types (*n*, %)	
*Frontal lobe epilepsy*	14 (42.4)
*Temporal lobe epilepsy*	12 (36.4)
*Unspecified genetic generalized epilepsy*	1 (3.0)
*Other*	6 (18.2)
Predominant seizure types (*n*, %); and seizure frequency (mean ± SD) at baseline	
*Focal aware seizure*	4 (12.1); 6.9 ± 16.7
*Focal impaired awareness seizure*	21 (63.6); 4.2 ± 6.4
*Focal to bilateral tonic–clonic seizure*	7 (21.2); 0.2 ± 0.7
*Seizure free*	1 (3.0)
Number of ASMs at baseline (*n*, %)	
*1*	1 (3.05)
*2*	14 (42.4)
*3*	17 (51.5)
*4*	1 (3.05)
VNS model (*n*, %)	
*1000 (Sentiva)*	10 (30.3)
*106 (Aspire)*	23 (69.7)
Duration of VNS therapy (median, (range))	29 (12 to 60)

Abbreviations: IQR = interquartile range, AEF = attention and executive functions, ASM = antiseizure medications.

### 2.4 Cognitive Evaluation

The patients were assessed with the maze, interference, and written verbal fluency tests according to our standard clinical VNS protocol as a part of EpiTrack testing. In the maze test, patients are asked to track a maze like driving a car (Chapuis 1992). In the interference test, subjects are required to read three rows of ones and twos in reverse order (e.g., reading 11212 as 22121) (Lehrl and Fisher 1997). In the written phonemic verbal fluency task, subjects are asked to write down as many words as possible within a 60‐s timeframe that begins with a designated letter (Horn 1983). The object of evaluation in the maze and interference tests is the time needed to perform the tasks, while in the written verbal fluency test, the number of words produced is observed.

EpiTrack subtest scores range from 1 to 7 points, with 1 point indicating the impaired end of performance. However, the scores of the EpiTrack subtests represent only ordinal indices and should not be interpreted as interval‐scaled scores (Lutz and Helmstaedter 2005). Clinically meaningful improvement in test performance was defined as a change in raw performance ≥ the range within the subtest score (5 s for the interference test, 15 s for the maze test and 6 words on the verbal fluency test).

### 2.5 Statistical Analysis

Changes in the interference, maze and verbal fluency scores over time (months) were analysed using a LME model with robust standard errors in Stata version 17.0 (StataCorp, College Station, Texas, USA). The outcome variables were three cognitive test scores (continuous), and the exposure variables were clinical characteristics (psychiatric comorbidities, epilepsy types, predominant seizure types, and ASMs) and time (continuous, in months). Visual representations of the results include observed values of the scores for each test at each time point and fitted average trajectories based on LME models. In addition, the changes in the scores for each test over a follow‐up period of up to 5 years are represented by the estimates (with 95% confidence intervals) predicted by the model. *p* values ≤ 0.05 were considered significant. Since the LME model does not incorporate changes in ASMs or seizure frequency during VNS therapy, we performed an additional descriptive analysis to demonstrate changes in relevant clinical features at the individual patient level.

## 3 Results

Average baseline scores in the three tests for the whole study population and in different clinical factors are presented in Table 2.

**TABLE 2:** Average baseline scores and change per month for all patients as well as in different clinical categories based on linear mixed effects models.

Clinical parameters	*N* (%)	Maze test score (s)				Interference test score (s)				Verbal fluency test score (words)			
		**Baseline**	**Change (s/month)**	**95% CI**	***p*** Value	**Baseline**	**Change (s/month)**	**95% CI**	***p*** Value	**Baseline**	**Change (number of words/month)**	**95% CI**	***p*** Value
All patients	33 (100)	34.5	−0.20	−0.365 to −0.041	**0.014**	22.5	−0.05	−0.131 to 0.028	0.207	9.8	0.030	−0.003 to 0.064	0.079
Psychiatric comorbidity													
*No*	24 (72.7)	30.64	−0.06	−0.249 to 0.123	0.534	20.79	−0.03	−0.135 to 0.069	0.534	10.83	0.02	−0.021 to 0.062	0.337
*Yes*	9 (27.3)	42.63	−0.52	−0.828 to −0.206	**0.001**	26.58	−0.11	−0.264 to 0.039	0.147	7.26	0.05	−0.017 to 0.123	0.138
Epilepsy type													
*FLE*	14 (42.4)	30.31	−0.17	−0.381 to 0.033	0.099	23.82	−0.14	−0.237 to −0.043	**0.005**	10.6	0.03	−0.019 to 0.075	0.245
*TLE*	12 (36.4)	38.42	−0.25	−0.517 to 0.017	0.067	21.18	−0.01	−0.074 to 0.062	0.867	7.45	0.07	0.016 to 0.1232	**0.011**
*Other*	7 (21.2)	35.91	−0.16	−0.607 to 0.286	0.481	21.71	0.08	−0.219 to 0.369	0.615	12.11	−0.05	−0.111 to 0.0177	0.155
Predominant seizure type													
*FAS/FIAS*	25 (75.8)	34.68	−0.19	−0.413 to 0.029	0.089	21.23	−0.03	−0.125 to 0.066	0.540	10.17	0.03	−0.011 to 0.079	0.137
*FBTCS*	8 (24.2)	33.66	−0.25	−0.544 to 0.040	0.091	27.72	−0.16	−0.353 to 0.024	0.087	8.86	0.02	−0.037 to 0.082	0.461
ASMs													
*1*–*2*	15 (45.5)	25.66	−0.22	−0.409 to −0.032	**0.022**	22.04	−0.14	−0.264 to −0.011	**0.033**	11.44	0.04	−0.0097 to 0.099	0.108
*3*–*4*	18 (54.5)	41.08	−0.19	−0.468 to 0.085	0.175	22.71	0.007	−0.097 to 0.112	0.889	8.54	0.02	−0.033 to 0.066	0.517

*Note*: Bold values indicate statistically significant *p* values.

Abbreviations: CI = confidence interval, FLE = frontal lobe epilepsy, TLE = temporal lobe epilepsy, FAS = focal aware seizure, FBTCS = focal to bilateral tonic–clonic seizure, FIAS = focal impaired awareness seizure, ASM = antiseizure medication.

### 3.1 Changes in Interference, Maze and Verbal Fluency Scores During Follow‐Up

The median duration of VNS after implantation was 29 months and ranged from 12 to 60 months. The stimulation parameters used in the patients included a current range of 1 mA to 1.75 mA, a frequency range of 20 Hz to 30 Hz, and an off‐time range of 1.1 to 5 min. All patients had an on‐time of 30 s and a pulse width of 250 µs.

**FIGURE 1:**
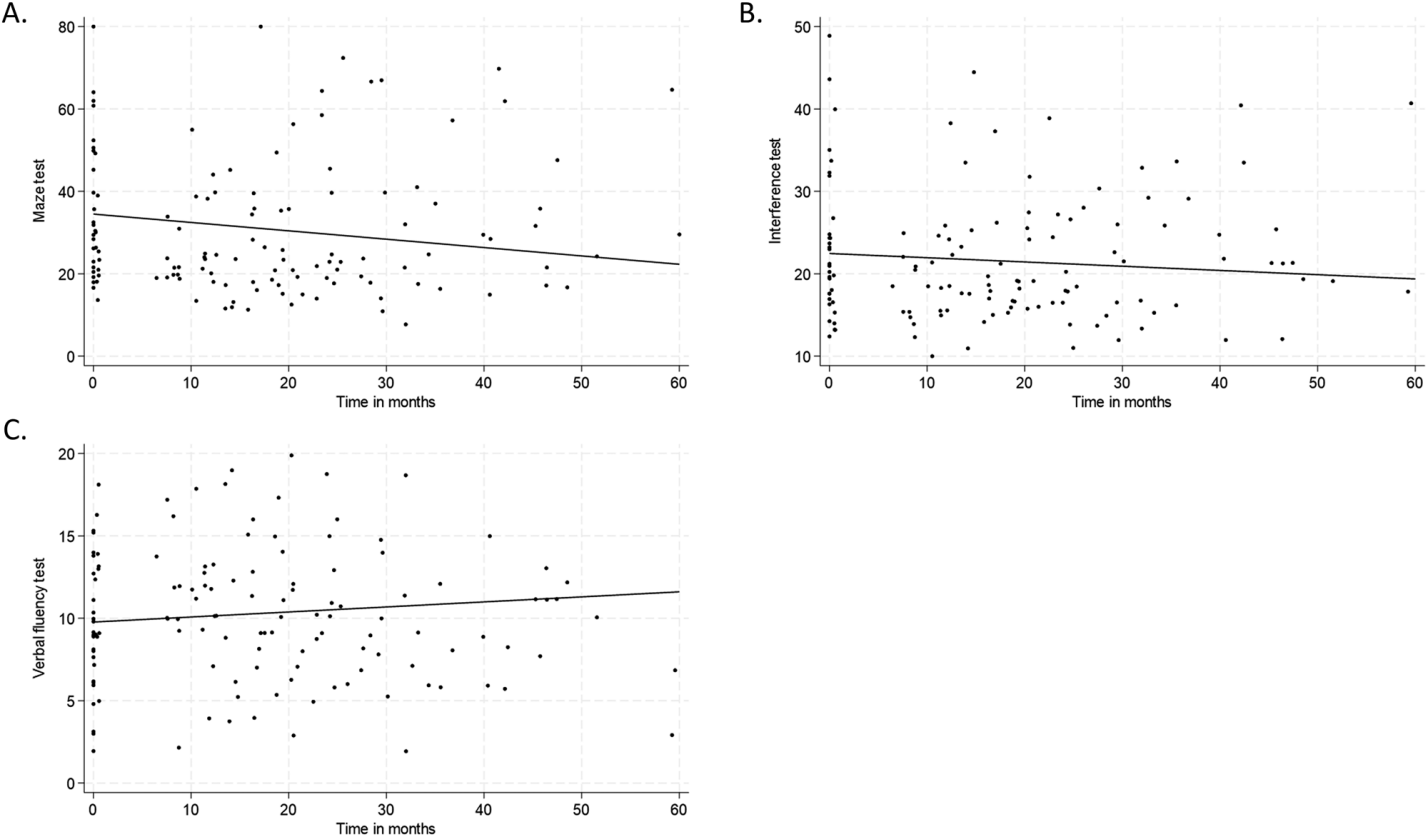
Observed (A) maze, (B) interference and (C) verbal fluency test scores and fitted curve based on linear mixed‐effects model for all patients over time following VNS therapy. The baseline scores for interference, maze and verbal fluency were 34.5 s, 22.5 s and 9.8 words, respectively.

During the follow‐up, maze test time improved significantly by an average of 0.20 s per month (*p* = 0.014), interference test time improved by an average of 0.05 s per month (*p* = 0.207) and verbal fluency test improved by an average of 0.03 words per month (*p* = 0.079) (Figure 1 and Table 2). In the maze test, the average improvement was 12 s, and the corresponding EpiTrack subtest score changed from a baseline score of 5 to 6 at 5 years. In terms of interference and verbal fluency, the corresponding EpiTrack subtest scores did not change during the 5‐year follow‐up.

At group level, patients with severely impaired baseline AEF performance improved significantly on the interference test during follow‐up (0.11 s/month; *p* = 0.028), but not on the maze or verbal fluency tests (Table 3).

**TABLE 3:** Number of patients displaying clinically meaningful improvement in different baseline AEF performance categories and average test score change per month in maze, interference, and verbal fluency scores depending on the baseline performance category.

Tests	Patients with clinically meaningful improvement	Changes in test scores (s or words/month)			
	***N* (%)**	**Average change**	**95% CI**	***p* Value**	**Number of patients and (testsNo)**
**Maze**					
All patients	8/33 (24%)	−0.20	−0.364 to –0.041	**0.014**	33 (128)
*Normal*	0 /12 (0%)	−0.14	−0.277 to −0.009	**0.036**	12 (44)
*Mild impairment*	2/8 (25%)	−0.28	−0.665 to 0.103	0.152	8 (31)
*Severe impairment*	6/13 (46%)	−0.21	−0.492 to 0.073	0.146	13 (53)
**Interference**					
All patients	6/33 (18%)	−0.05	−0.131 to 0.028	0.207	33 (130)
*Normal*	1/12 (8%)	−0.04	−0.095 to 0.022	0.225	12 (44)
*Mild impairment*	0/8 (0%)	0.08	−0.114 to 0.274	0.418	8 (31)
*Severe impairment*	5/13 (38%)	−0.11	−0.225 to −0.013	**0.028**	13 (55)
**Verbal fluency**					
All patients	3/33 (9%)	0.03	−0.003 to 0.064	0.079	33 (130)
*Normal*	0 /12 (0%)	0.04	−0.021 to 0.100	0.201	12 (44)
*Mild impairment*	0/8 (0%)	−0.02	−0.060 to 0.021	0.347	8 (31)
*Severe impairment*	3 /13 (23%)	0.06	−0.001 to 0.110	0.054	13 (55)

*Note*: Bold values indicate statistically significant *p* values.

Abbreviations: AEF = attention and executive functions, CI = confidence interval.

Individual changes on the maze, interference, and verbal fluency tests scores as well as changes in ASM use and seizure frequency during the follow‐up period are presented in Tables S1–S3. The greatest proportion of patients achieved clinically meaningful improvement on the maze test, followed by the interference and verbal fluency tests (24%, 18% and 9%, respectively) (Table 3). Furthermore, among the patients with severely impaired AEF performance at baseline, 46% on the maze test, 38% on the interference test, and 23% on the verbal fluency test exhibited clinically meaningful improvements during the follow‐up. In contrast, among patients with normal baseline AEF performance, only 8% experienced clinically meaningful improvement on the interference and none on the maze or verbal fluency test.

### 3.2 Effect of Psychiatric Comorbidities on Interference, Maze and Verbal Fluency Performance

During the follow‐up, patients with psychiatric comorbidities exhibited significantly improved performance on the maze test (on average 0.52 s/month, *p* = 0.001), with no significant changes observed on the interference and verbal fluency tests (Table 2 and Figure S2). Maze performance improved by 31.2 s, and the corresponding EpiTrack subtest score changed from a baseline score of 5 to 7 at 5 years for patients with psychiatric comorbidities. In contrast, patients without psychiatric comorbidities did not experience notable improvements on any of the tests (Table 2).

### 3.3 Effect of Epilepsy Type on Interference, Maze and Verbal Fluency Performance

During the follow‐up, interference performance improved significantly for patients with FLE (0.14 s/month, *p* = 0.005), whereas no change was noted in patients with TLE or other types of epilepsy (Table 2 and Figure S3). For patients with FLE, interference performance improved by 8.4 s with the corresponding EpiTrack subtest score changing from a baseline score of 4 to 6 at 5 years. Improvement on the maze was similar for all the groups. Verbal fluency improved significantly for patients with TLE (0.07 words/month, *p* = 0.011) but not for patients with FLE or other types of epilepsy (Table 2 and Figure S3).

### 3.4 Effect of Predominant Seizure Type on Interference, Maze and Verbal Fluency Performance

During the follow‐up, improvement on the interference test was greater in the FBTCS group with the corresponding EpiTrack subtest score changing from a baseline score of 4 to 6 at 5 years than in the FAS/FIAS group (0.16 s/month, *p* = 0.087; 0.03 s/month, *p* = 0.540, respectively). Performance on the maze test improved similarly for both the FAS/FIAS and FBTCS groups without reaching statistical significance (0.19 s/month, *p* = 0.089; 0.25 s/month, *p* = 0.091, respectively) (Table 2 and Figure S4). Since we had only categorical classification of seizure responses, a statistical analysis of the effect of seizure frequency change on cognitive test performances was not feasible.

Furthermore, among the seizure responders (≥ 50% reduction), 20% exhibited clinically meaningful improvement on the maze test, 13% on the interference test, and 13% on the verbal fluency test during the follow‐up. In comparison, among the patients who experienced clinically meaningful improvements on these tests, 37.5% on the maze test, 33% on the interference test, and 67% on the verbal fluency test were responders for their predominant seizure type. Within the FBTCS group, none of the patients who showed clinically meaningful improvement on interference were seizure responders, whereas in the FAS/FIAS group, 50% of the patients who showed clinically meaningful improvement were seizure responders (Tables S1–S3).

### 3.5 Effect of ASMs on Interference, Maze and Verbal Fluency Performance

During the follow‐up, interference test time improved significantly for patients taking 1–2 ASMs (0.14 s/month, *p* = 0.033), while that of patients taking 3–4 ASMs did not improve. On the maze test, improvements were similar for patients taking 1–2 ASMs and for those taking 3–4 ASMs (0.22 s/month, *p* = 0.022; 0.19 s/month, *p* = 0.175, respectively), but statistical significance was found only for patients taking 1–2 ASMs (Table 2 and Figure S5).

During the follow‐up, 48% of patients had a reduction in the ASM burden. Among these patients, 44% showed a clinically meaningful improvement on the maze test, 25% on the interference test, 6% on the verbal fluency test, and none on all three tests (Tables S1–S3).

## 4 Discussion

The main finding of our study was that among the three cognitive tests assessing different aspects of AEFs, performance on the maze test improved, whereas on the interference and verbal fluency tests, notable changes were not observed at the group level in DRE patients receiving VNS therapy for up to 5 years. However, significant differences in changes in test performance related to specific clinical features were observed. First, among patients with psychiatric comorbidities performance on the maze test improved by far the most. Additionally, patients with FLE and those taking 1–2 ASMs improved significantly in interference test, and patients with FBTCS exhibited a similar degree of change without achieving statistical significance. Finally, on the verbal fluency test, baseline performance was markedly worse than on the other two tests, and clinically meaningful improvement was not observed in any of the groups during the follow‐up, even though patients with TLE showed statistically significant improvement.

On all three tests but particularly on the interference, the possibility of achieving clinically meaningful improvement was greatest if baseline AEFs performance was severely impaired, which is consistent with our previous findings (Lähde et al. 2024b; Lähde et al. 2024a). In the whole study population, the LME model predicted a change of 0.20 s per month on the maze test, resulting in an improvement of 12 s at 5 years, with the corresponding EpiTrack subtest score changing from a baseline score of 5 to 6. This finding is in line with the results of two previous studies investigating EpiTrack total score changes in the same study population (Lähde et al. 2024b) as well as changes on the Trail‐Making Test and Digit Span Backward Task in an extended study population during VNS therapy, demonstrating significant improvements on all three tests at the group level (Lähde et al. 2024a). However, contrary to previous results, in this study, performance on the verbal fluency and interference tests did not improve significantly during the follow‐up. The maze task specifically measures visual anticipation while also imposing demands on planning and psychomotor speed, the interference test focuses on evaluating response inhibition, and performance on the verbal fluency test assesses verbal abilities along with executive control (Lutz and Helmstaedter 2005; Shao et al. 2014). There is some experimental evidence on physiological effects of VNS on visual processing. A recent sham‐controlled study of the effect of cervical transcutaneous VNS on sensory performance in neurotypical adults improved visual performance, which was attributed to locus coeruleus‐norepinephrine‐mediated suppression of calcium T‐type channels responsible for bursting activity by sensory relay neurons in the thalamus that reduces the accuracy and efficiency of sensory transmission (Jigo et al. 2024). These findings suggest that visual anticipation is more susceptible to the positive effects of VNS therapy than are response inhibition or verbal abilities.

When evaluating the significance of psychiatric comorbidities on both baseline performance and performance changes on these three tests, we observed that baseline performance was worse on all three tests for patients with psychiatric comorbidities than for those without. Moreover, patients with psychiatric comorbidities showed significant improvement on the maze test during the follow‐up, which was notably stronger than the observed changes in the whole study population or in patients without psychiatric comorbidities. The predicted improvement on the maze test in the group of patients with psychiatric comorbidities was 31.2 s at 5 years, with the corresponding EpiTrack subtest score changing from a baseline score of 5 to 7. However, changes in interference and verbal fluency did not differ between patients with or without psychiatric comorbidities during the follow‐up. It is also possible that psychiatric medications have changed during the follow‐up influencing the results. Additionally, the small sample size in the psychiatric comorbidities’ subgroup presents its own limitations. Finally, due to the multitude of both direct and indirect effects of VNS therapy on cognitive functioning, disentangling the effect of individual factors is not possible in a real‐world setting.

Interestingly, when addressing epilepsy types, patients with FLE markedly improved on the interference test, while those with TLE and other types of epilepsy remained unchanged. The enhancement detected on the interference test among FLE patients might be explained by the VNS‐induced augmentation of frontal networks, which are potentially more compromised in FLE patients than in TLE patients or patients with other types of epilepsy (Widjaja et al. 2015). Furthermore, frontal lobe functions are specifically crucial for response inhibition.

Poor seizure control often correlates with diminished cognitive performance (Dodril 2004). In the current study, we mainly focused on evaluating the impact of seizure type on AEFs, rather than the effect of seizure frequency. During the follow‐up, patients with FBTCS improved their interference performance by 9.6 s, with the corresponding EpiTrack subtest score changing from a baseline score of 4 to 6, while patients with FAS/FIAS did not improve from a baseline score of 5. On the maze and verbal fluency tests, both baseline performance and changes in performance during the follow‐up were almost identical for both groups. Apparently, response inhibition seems to be particularly sensitive to the negative effects of FBTCS on cognition. Interestingly, within the FBTCS group, none of the patients who showed clinically meaningful improvement in interference were seizure responders, whereas in the FAS/FIAS group, 50% were seizure responders. This finding implies that the improvement in interference among FBTCS patients during VNS therapy was not dependent solely on decreased seizure frequency.

The detrimental impact of using more than two ASMs on AEFs has been established in several previous studies (Witt, Elger, and Helmstaedter 2013, Witt, Elger, and Helmstaedter 2015). In the present study, patients taking 1–2 ASMs performed significantly better on the maze and verbal fluency tests at baseline than those taking 3–4 ASMs. However, performance on the interference test was similar for both groups. This observation suggests that a high number of concurrent ASMs does not exert as detrimental impact on response inhibition as on verbal fluency, visual anticipation or processing speed. During the follow‐up, patients taking 1–2 ASMs improved significantly on the interference test, whereas patients taking 3–4 ASMs did not exhibit any improvement. Changes in maze and verbal fluency performance did not display notable differences between patients with 1–2 and 3–4 ASM, although only the improvement for patients taking 1‐ 2 ASMs was significant.

The main limitation of our study is its retrospective and uncontrolled design, along with the analysis of the data collected in accordance with the clinical protocol. Moreover, practical limitations in assessing patients within a single‐centre setting contributed to the restricted sample size; therefore, attaining statistical significance was challenging, especially in subgroup analyses. Due to the COVID‐19 pandemic, the scheduled visits did not always occur according to our clinical VNS follow‐up protocol. Therefore, changes in the test scores over time were analysed using a statistical model to compensate for variations in follow‐up time points and the numbers of tests administrated to individual patients when predicting result changes per month during a period of up to 5 years. Furthermore, the LME model did not incorporate potential adjustments to ASMs, fluctuations in seizure status, or variations in the severity of depression during the follow‐up period. On the other hand, employing the LME model provided a statistically robust evaluation of the test scores as time series data following VNS implantation.

Finally, cognitive tests are vulnerable to significant practice effects in repeated testing sessions. Several factors influence practice effects, with the length of the test–retest interval being particularly crucial (Calamia, Markon, and Tranel 2012; Scharfen, Peters, and Holling 2018). A longer test–retest interval between each administration corresponds to the lower practice effects. A plateau in improvement seems to be reached after the administration of the third test. All patients in our study underwent a minimum of three assessments. Moreover, using parallel forms of cognitive tests may attenuate practice effects (Calamia, Markon, and Tranel 2012). Accordingly, the improvements observed in the retest scores in this study may have been influenced by practice effects, which should be considered when interpreting the results. However, for the maze and verbal fluency tests, retest versions were available to minimize potential practice effects. Consequently, the more pronounced improvement on the maze test compared to that on the two other tests is unlikely due to practice effects.

## 5 Conclusions

Following VNS therapy, a gradual and significant improvement in the maze test was observed at the group level, while performance on the verbal fluency and interference tests remained relatively stable. Consequently, visual anticipation and planning displayed superior enhancement compared to response inhibition and verbal fluency during VNS therapy. Furthermore, patients with psychiatric comorbidities exhibited even greater improvement on the maze performance. The performance variability observed in these tree cognitive tests supports the importance of evaluating specific tests separately.

## Author Contributions

**Niina Lähde**: conceptualization; writing–original draft; writing–review and editing; investigation. **Pabitra Basnyat**: investigation; data curation; writing–review and editing. **Jani Raitanen**: methodology; software; formal analysis; writing–review and editing. **Leena Kämppi**: writing–review and editing; investigation. **Kai Lehtimäki**: conceptualization; investigation; writing–review and editing; visualization; supervision. **Eija Rosti‐Otajärvi**: conceptualization; investigation; writing–review and editing; visualization; supervision. **Jukka Peltola**: conceptualization; investigation; writing–review and editing; validation; supervision; funding acquisition.

## Ethics Statement

This was a non‐interventional study in which data was collected prospectively but analysed retrospectively from a VNS quality register at Tampere University Hospital, therefore, not requiring ethics committee approval according to Finnish Law onResearch.

## Conflicts of Interest

Niina Lähde has participated in a clinical trial for UCB; received speaker´s honoraria from LivaNova (OmaMedical). Leena Kämppi has received speaker´s honoraria from UCB, Merck, and Eisai; received support for travel to congress from UCB and Angelini Pharma. Kai Lehtimäki has received speaker´s honoraria from Medtronic. Eija Rosti‐Otajärvi has received speaker´s honoraria from Novartis and Biogen. Jukka Peltola has participated in clinical trials for Eisai, UCB, and Bial; received research grants from Angelini Pharma, Eisai, Medtronic, UCB, and LivaNova; received speaker´s honoraria from LivaNova, Angelini Pharma, Eisai, Jazz Pharma, Medtronic, Orion Pharma, and UCB; received support for travel to congresses from LivaNova, Eisai, Medtronic, and UCB; and participated in advisory boards for LivaNova, Angelini Pharma, Jazz Pharma, Eisai, Medtronic, UCB, and Pfizer. The remaining authors have no conflicts of interest.

### Peer Review

The peer review history for this article is available at https://publons.com/publon/10.1002/brb3.70176.

## Supporting information



Supplementary Materials.

## Data Availability

The data that support the findings of this study are available on request from the corresponding author. The data are not publicly available due to privacy or ethical restrictions.
