# Supplementary material for: Changes in Response Inhibition, Visual Anticipation and Verbal Fluency During Vagus Nerve Stimulation Therapy in Patients With Drug‐Resistant Epilepsy
Source: Brain Behav. 2024 Dec 6;14(12):e70176. doi: 10.1002/brb3.70176 (PMC11624008; doi:10.1002/brb3.70176)
Supplement: Supplementary file 1 — Supplementary Materials. [file BRB3-14-e70176-s001.pdf]

Supplementary Table 1. Individual changes on ASMs, seizure frequency and test scores during follow-up period in patients with **normal** performance in EpiTrack at baseline.

Sex / Age at baseline	Epilepsy type	Psych.com.	ASMs at baseline	ASMs at final observation point	Predominant seizure type	Monthly seizure frequency 12 months prior baseline	Change in predominant seizure type compared to baseline	Duration of stimulation (months)	EpiTrack change	Change in maze seconds (subtest score)	Change in Interference seconds (subtest score)	Change in Verbal fluency words (subtest score)
M / 53	Other	No	LCM 400, LEV 2000	LCM 400, LEV 2000	FIAS	10	↓ < 25%	13	38 → 40	14 → 15 (7 → 7)	20 → 19 (5 → 5)	14 → 14 (4 → 4)
F / 44	TLE	No	BRV 200, LCM 400	BRV 200, LCM 400	FAS	54	↓ < 25%	24	36 → 37	27 → 20 (6 → 6)	18 → 17 (6 → 6)	14 → 19 (4 → 4)
F / 33	FLE	No	LTG 500, TPM 400	LTG 500, TPM 50↓	FIAS	0.3	↓ 100 %	14	34 → 36	25 → 12 (6 → 7)	13 → 11 (7 → 7)	18 → 19 (4 → 4)
M / 22	TLE	No	BRV 200, ESL 1600	BRV 200, ESL 1600	FIAS	12	↓ 100 %	26	33 → 35	16 → 15 (7 → 7)	16 → 17 (6 → 6)	9 → 9 (3 → 3)
M / 32	FLE	No	LEV 2000, ESL 2000	BRV 200, ESL 2000	FIAS	3	↓ ≥ 75% < 90%	29	32 → 37	19 → 11 (6 → 7)	15 → 12 (6 → 7)	13 → 14 (3 → 4)
F / 32	FLE	No	LEV 3000, LCM 600	LEV 3000, LCM 600, CLB 5 +	FBTCS	0.3	No change	29	32 → 33	18 → 16 (6 → 7)	23 → 16 (5 → 6)	14 → 12 (4 → 3)
F / 26	Other	No	LEV 3000, LCM 500	LEV 3000, LCM 500	FIAS	0.5	No change	32	32 → 33	21 → 20 (6 → 6)	14 → 16 (6 → 6)	13 → 11 (3 → 3)
F / 31	TLE	YES / past	LCM 400, CLB 50	LCM 400, CLB 35↓, BRV 150 +	FIAS	8	↓ ≥ 25 < 50%	48	32 → 32	20 → 24 (6 → 6)	20 → 19 (5 → 5)	8 → 10 (3 → 3)
F / 24	TLE	No	LTG 500, LEV 1750, ZNS 200	LTG 500, LEV 1000↓, ZNS%	FIAS	1	↑ 100 %	33	33 → 32	30 → 18 (6 → 6)	18 → 14 (6 → 6)	9 → 13 (3 → 3)
M / 29	FLE	No	OXC 2100, ZNS 500, CLB 20	OXC 2100, ZNS 50↓, CLB 20	SF	SF	No change	29	33 → 33	23 → 18 (6 → 6)	13 → 15 (7 → 6)	9 → 9 (3 → 3)
M / 29	FLE	No	LCM 600, PER 8, CLB 20	LCM 600, PER 8, CLB 20	FBTCS	0,3	↓ 100%	12	32 → 33	20 → 21 (6 → 6)	18 → 16 (6 → 6)	10 → 15 (3 → 4)
F / 22	Other	No	ESL 1600, CLB 15, BRV 200	ESL 1600, CLB 15, BRV 200	FIAS	6	↓ ≥ 25 < 50%	12	34 → 33	17 → 17 (7 → 7)	16 → 18 (6 → 6)	16 → 13 (4 → 3)

Abbreviations: ↓= *decrease/reduction,* ↑ = *increase,* + = *add on,* % = *withdrawn*, ASM = antiseizure medication, BRE = brivaracetam, CLB = clobazam, ESL = eslicarbazepine acetate, LCM = lacosamide, LEV = levetiracetam, LTG = lamotrigine, OXC = oxcarbamazepine, PER = perampanel, TLE = temporal lobe epilepsy, TPM = topiramate, ZNS = zonisamide, FAS = focal aware seizure, FBTCS = focal to bilateral tonic clonic seizure, FIAS = focal impaired awareness seizure, FLE =frontal lobe epilepsy, TLE = temporal lobe epilepsy. The green boxes in the ' ASMs at final observation point' column highlights ASM decrease/reduction, while the red boxes indicate ASM increase. In the ‘Change in predominant seizure type compared to baseline' column, green highlights more than 50% seizure reduction, and red indicates more than 50% seizure increase. In the 'EpiTrack change' column and in the subsequent columns, green highlights patients with clinically significant improvement.

Supplementary Table 2. Individual changes on ASMs, seizure frequency and test scores during follow-up period in patients with **mildly impaired** performance in EpiTrack at baseline.

Sex / Age at baseline	Epilepsy type	Psych.com.	ASMs at baseline	ASMs at final observation point	Predominant seizure type	Monthly seizure frequency 12 months prior baseline	Change in predominant seizure type compared to baseline	Duration of stimulation (months)	EpiTrack change	Change in maze seconds (subtest score)	Change in Interference seconds (subtest score)	Change in Verbal fluency words (subtest score)
M / 39	FLE	Yes / past	LCM 350, CBZ 1100	LCM 400↑, CBZ 1100	FIAS	7	↓ ≥ 50% < 75%	20	30 -> 34	33 → 23 (5 → 6)	20 → 18 (5 → 6)	13 → 15 (3 → 4)
F / 32	TLE	Yes/ current	LTG 400, TPM 450	LTG 400, TPM 325↓	FBTCS	NA	NA	40	29 -> 30	28 → 26 (6 → 6)	21 → 22 (5 → 5)	6 → 7 (2 → 2)
F / 23	FLE	No	LEV 3000, LCM 500	LEV 3000, LCM 500	FBTCS	1	↓ 100%	25	29 -> 27	29 → 24 (6 → 6)	23 → 19 (5 → 5)	11 → 11 (3 → 3)
M / 33	TLE	Yes / current	ZNS 300, OXC 1800, PGB 225	ZNS 200↓, OXC 1800, PGB%	FAS	70	↓ ≥ 75% < 90%	35	31 -> 37	60 → 17 (4 → 7)	12 → 12 (7 → 7)	15 → 13 (4 → 3)
F / 28	FLE	No	LCM 600, BRV 200, PER 2	LCM 600, BRV 200, PER 4↑	FIAS	0.5	↓ 100%	12	31 -> 32	23 → 24 (6 → 6)	21 → 22 (5 → 5)	9 → 10 (3 → 3)
M / 50	Other	No	ESL 1600, LEV 1500, CLB 30	ESL 1600, LEV 1500, CLB 30	FIAS	4	↓ ≥ 75% < 90%	13	30 -> 26	44 → 33 (5 → 5)	19 → 19 (5 → 5)	15 → 11 (4 → 3)
M / 51	Other	No	LEV 1000, ESL 1200, PER 6	LEV 1000, ESL 1200, PER%	FAS	10	↑ > 100%	46	29 -> 32	51 → 31 (4 → 6)	23 → 21 (5 → 5)	10 → 11 (3 → 3)
M / 39	Other	No	ZNS 500, LTG 500, CLB 50	ZNS 500, LTG 400↓, CLB 40↓	FIAS	0.5	↑ ≥ 75% < 90%	40	29 -> 14	50 → 63 (4 → 3)	17 → 41 (6 → 1)	10 → 6 (3 → 2)

Abbreviations: ↓= *decrease/reduction,* ↑ = *increase, + = add on, % = withdrawn*, ASM = antiseizure medication, BRE, brivaracetam, CBZ = Carbamazepine, CLB = clobazam, ESL= eslicarbazepine acetate, LCM = lacosamide, LEV = levetiracetam, LTG = lamotrigine, OXC = oxcarbamazepine; PER = perampanel, PGB = pregabalin; TLE = temporal lobe epilepsy, TPM = topiramate; ZNS = zonisamide. FLE = frontal lobe epilepsy, TLE = temporal lobe epilepsy, NA = not available. The green boxes in the ' ASMs at final observation point' column highlights ASM decrease/reduction, while the red boxes indicate ASM increase. In the ‘Change in predominant seizure type compared to baseline' column, green highlights more than 50% seizure reduction, and red indicates more than 50% seizure increase. In the 'EpiTrack change' column and in the subsequent columns, green highlights patients with clinically significant improvement.

Supplementary Table 3. Individual changes on ASMs, seizure frequency and test scores during follow-up period in patients with **severely impaired** performance in EpiTrack at baseline.

Sex / Age at baseline	Epilepsy type	Psych.com.	ASMs at baseline	ASMs at final observation point	Predominant seizure type	Monthly seizure frequency 12 months prior baseline	Change in predominant seizure type compared to baseline	Duration of stimulation (months)	EpiTrack change	Change in maze seconds (subtest score)	Change in Interference seconds (subtest score)	Change in Verbal fluency words (subtest score)
F / 41	FLE	No	LCM 600	LCM 600	FIAS	17	↓ ≥ 50% < 75%	31	25 -> 35	29 → 9 (6 → 7)	33 → 14 (3 → 6)	12 → 19 (3 → 4)
F / 28	FLE	No	OXC 1200, ZNS 400	OXC 1200, ZNS 250↓	FIAS	2	↓ ≥ 50% < 75%	29	18 -> 24	26 → 28 (6 → 6)	35 → 22 (2 → 5)	8 → 6 (3 → 2)
F / 38	Other	YES / current	PER 6, CLB 20	PER 4↓, CLB 20	FBTCS	4	No change	15	14 -> 25	64 → 39 (3 → 5)	49 → 37 (1 → 2)	6 → 8 (2 → 3)
F / 19	TLE	YES / current	OXC 2100, ZNS 400, LEV 1000	OXC 2100, ZNS 200↓, LEV%	FIAS	5	↑ ≥ 25 % < 50 %	29	28 -> 32	32 → 16 (6 → 7)	14 → 19 (6 → 5)	3 → 12 (2 → 3)
F / 22	TLE	No	LCM 500, BRV 150, ZNS 200	LCM 500, BRV 200↑, ZNS%	FIAS	2	↓ ≥ 75% < 90%	18	26 -> 29	35 → 21 (5 → 6)	24 → 24 (4 → 4)	7 → 10 (2 → 3)
F / 41	TLE	No	LTG 200, ZNS 300, VPA 600	LTG 200, ZNS 300, VPA 600, CLB 10+	FIAS	1	↓ 100%	60	24 -> 26	62 → 58 (4 → 4)	21 → 18 (5 → 6)	3 → 9 (2 → 3)
M / 46	FLE	No	LTG 200, VPA 1200, TPM 400	LTG 200, VPA 1200, TPM 400	FBTCS	1	↓ ≥ 50% < 75%	46	22 -> 29	39 → 37 (5 → 5)	24 → 26 (4 → 4)	6 → 8 (2 → 3)
M / 24	FLE	No	OXC 1800, PER 12, CLB 90	OXC 1800, PER 12, CLB 80↓	FAS	30	↓ < 25%	31	22 -> 18	23 → 57 (6 → 4)	33 → 29 (3 → 3)	8 → 8 (3 → 3)
F / 70	FLE	No	LTG 400, PER 10, CLB 30	LTG 400, PER 8↓, CLB%	FIAS	10	↓ < 25%	42	21 -> 30	242 → 48 (1 → 4)	25 → 21 (4 → 5)	9 → 11 (3 → 3)
M / 32	TLE	No	LTG 400, ZNS 400, CLB 40	LTG 400, ZNS 400, CLB 20↓	FIAS	4	↓ ≥ 25% < 50%	35	20 -> 19	50 → 70 (4 → 3)	27 → 33 (4 → 3)	9 → 8 (3 → 3)
F / 45	TLE	Yes / past	LCM 400, TPM 400, BRV 200	LCM 400, TPM 100↓, BRV 200	FIAS	5	↓ ≥ 25% < 50%	24	20 -> 26	38 → 40 (5 → 5)	32 → 21 (3 → 5)	2 → 5 (2 → 2)
M / 32	TLE	Yes / current	LTG 400, ZNS 500, CLB 20	LTG 400, CLB%, ZNS%	FIAS	Daily	↓ 100%	53	11 -> 19	80 → 30 (2 → 6)	40 → 41 (1 → 1)	5 → 7 (2 → 2)
F / 33	FLE	Yes / past	ZNS 500, ESL 1600, CLB 30, LEV 1500	ZNS 500, ESL 1600, LEV%, CLB%	FBTCS	1	↑ 100%	32	12 -> 17	50 → 33 (4 → 5)	44 → 33 (1 → 3)	5 → 2 (2 → 2)

Abbreviations: ↓= *decrease/reduction*, ↑ = *increase*, + = *add on*, *% = withdrawn*, ASM = antiseizure medication, BRE = brivaracetam, CLB = clobazam, ESL = eslicarbazepine acetate, LCM= lacosamide, LEV = levetiracetam, LTG = lamotrigine, OXC = oxcarbamazepine, PER = perampanel, TPM = topiramate, VPA = valproate, ZNS = zonisamide. FAS = focal aware seizure, FBTCS = focal to bilateral tonic clonic seizure, FIAS = focal impaired awareness seizure, FLE =

frontal lobe epilepsy, TLE = temporal lobe epilepsy. The green boxes in the ' ASMs at final observation point' column highlights ASM decrease/reduction, while the red boxes indicate ASM increase. In the ‘Change in predominant seizure type compared to baseline' column, green highlights more than 50% seizure reduction, and red indicates more than 50% seizure increase. In the 'EpiTrack change' column and in the subsequent columns, green highlights patients with clinically significant improvement.

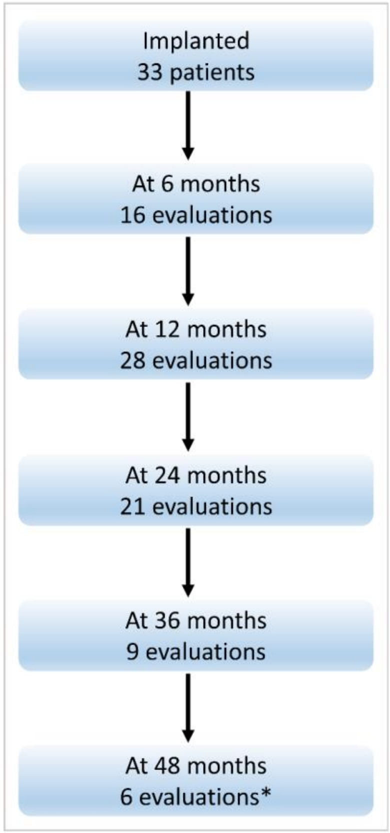

Supplementary Figure 1. The flowchart of the follow-up protocol. The figure includes summary of Maze, Interference and Verbal Fluency evaluations at different time points after VNS implantation. Each time point represents a specific duration in months as follows: 6 months (ranging from 4 to 8 months), 12 months (ranging from 18 to 30 months), 24 months (ranging from 30 to 42 months), and 36 months (ranging from 42 to 54 months). * Two evaluations at 61 and 70 months are included in this time point.

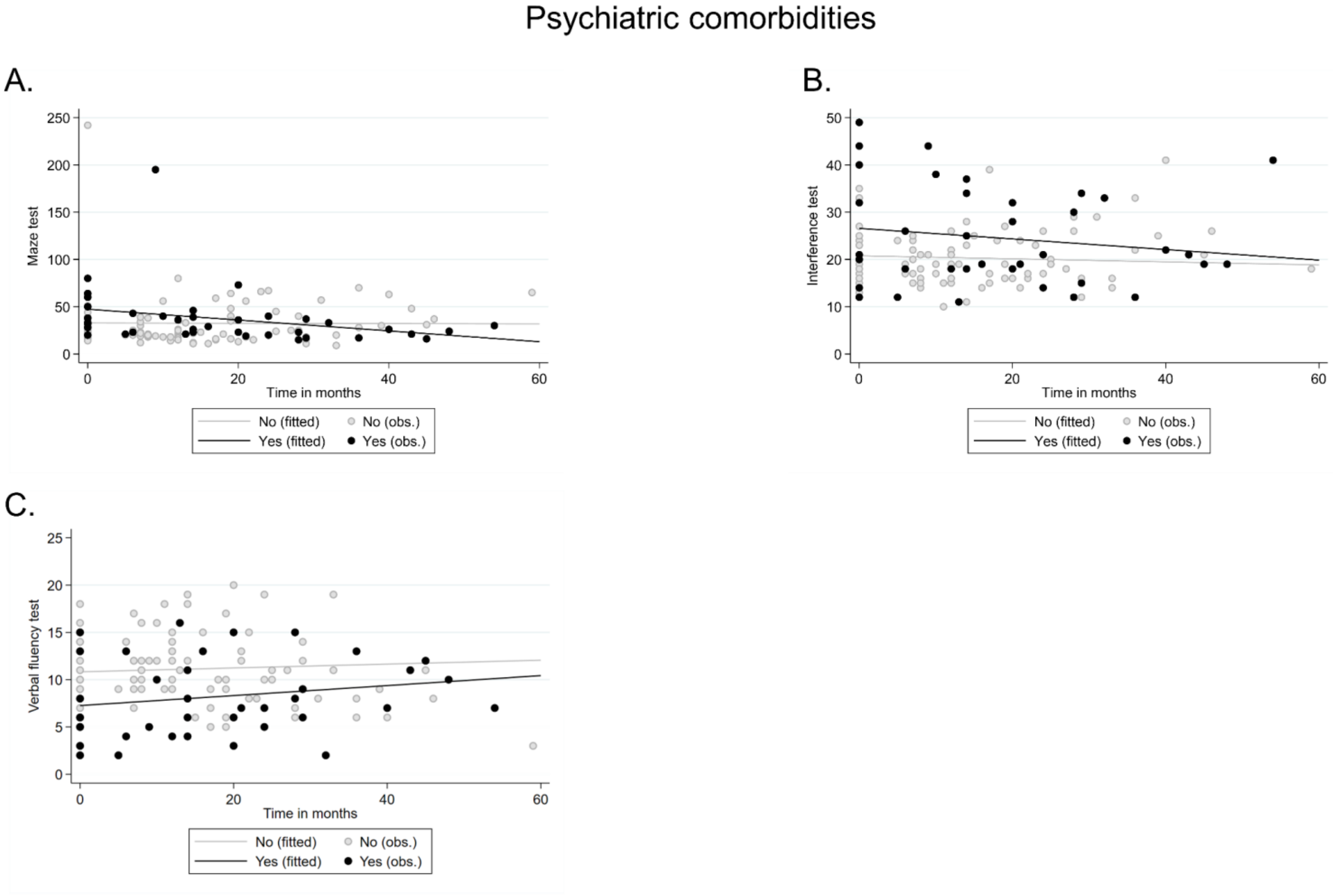

Supplementary Figure 2. Observed and fitted curves for **(A)** maze, **(B)** interference and **(C)** verbal fluency test scores based on linear mixed-effects model over time following VNS therapy based on presence of psychiatric comorbidities.

Epilepsy types

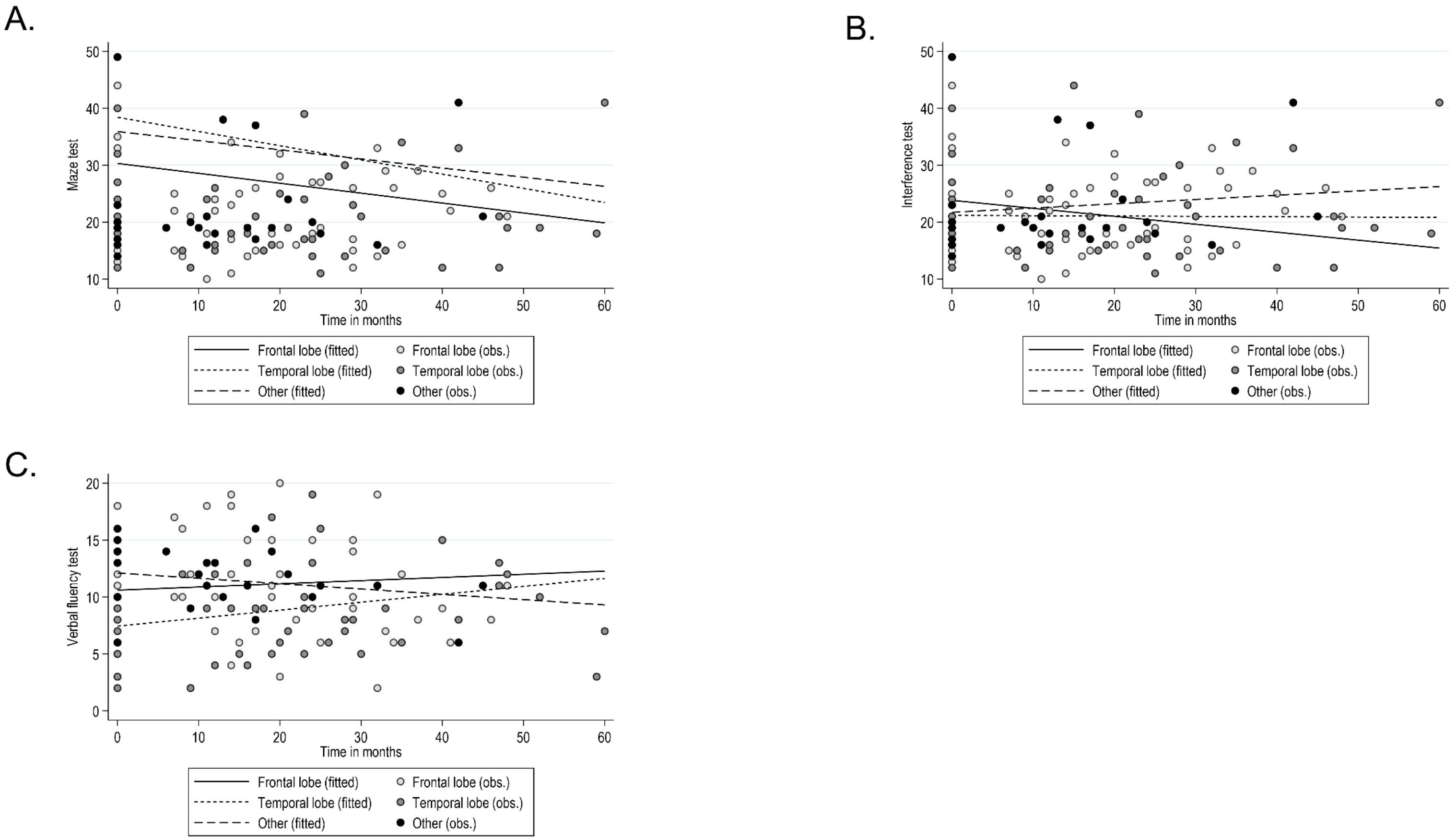

Supplementary Figure 3. Observed and fitted curves for **(A)** maze, **(B)** interference and **(C)** verbal fluency test scores based on linear mixed-effects model over time following VNS therapy based on the epilepsy types.

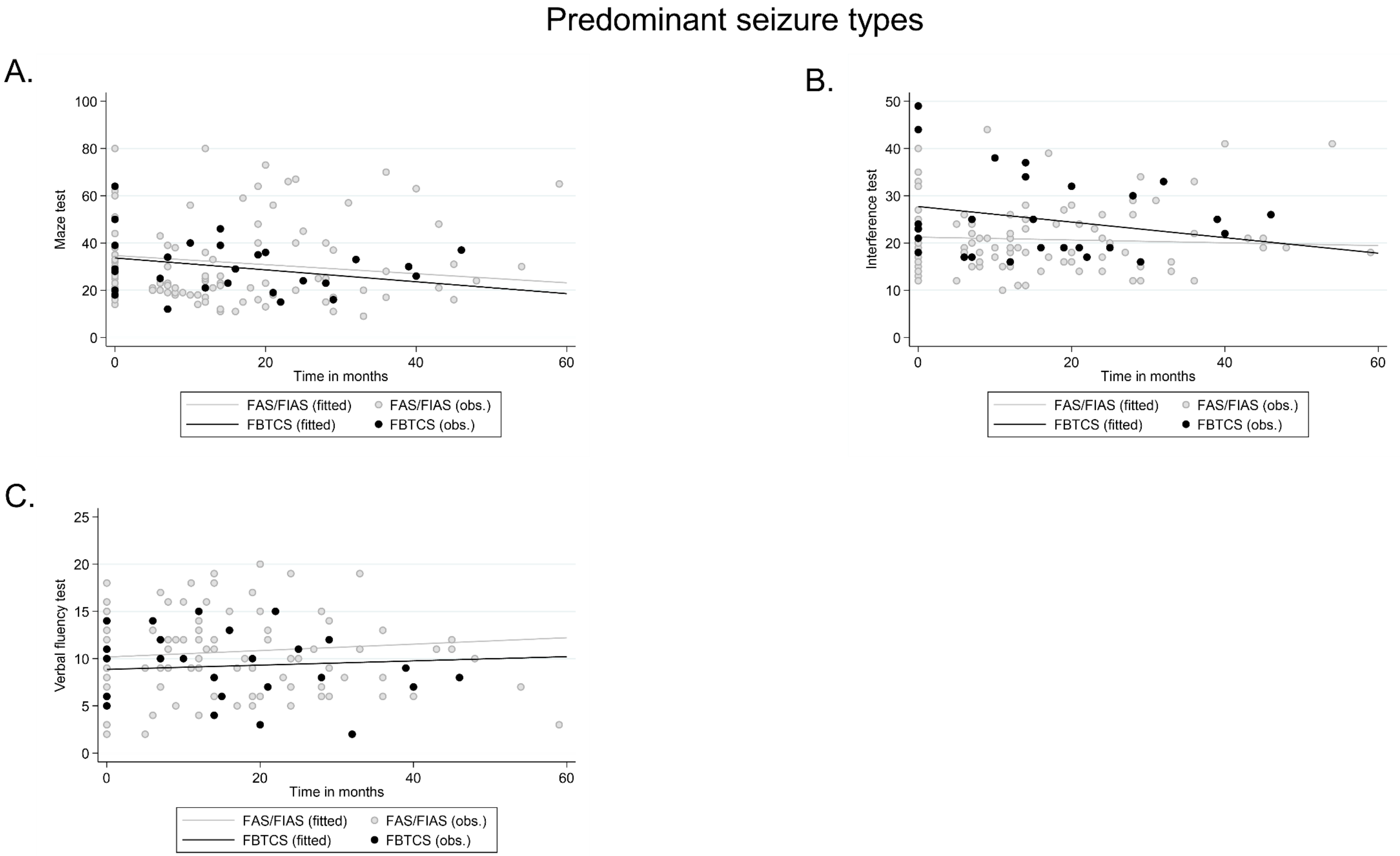

Supplementary Figure 4. Observed and fitted curves for **(A)** maze, **(B)** interference and **(C)** verbal fluency test scores based on linear mixed-effects model over time following VNS therapy based on the predominant seizure types.

Antiseizure medications

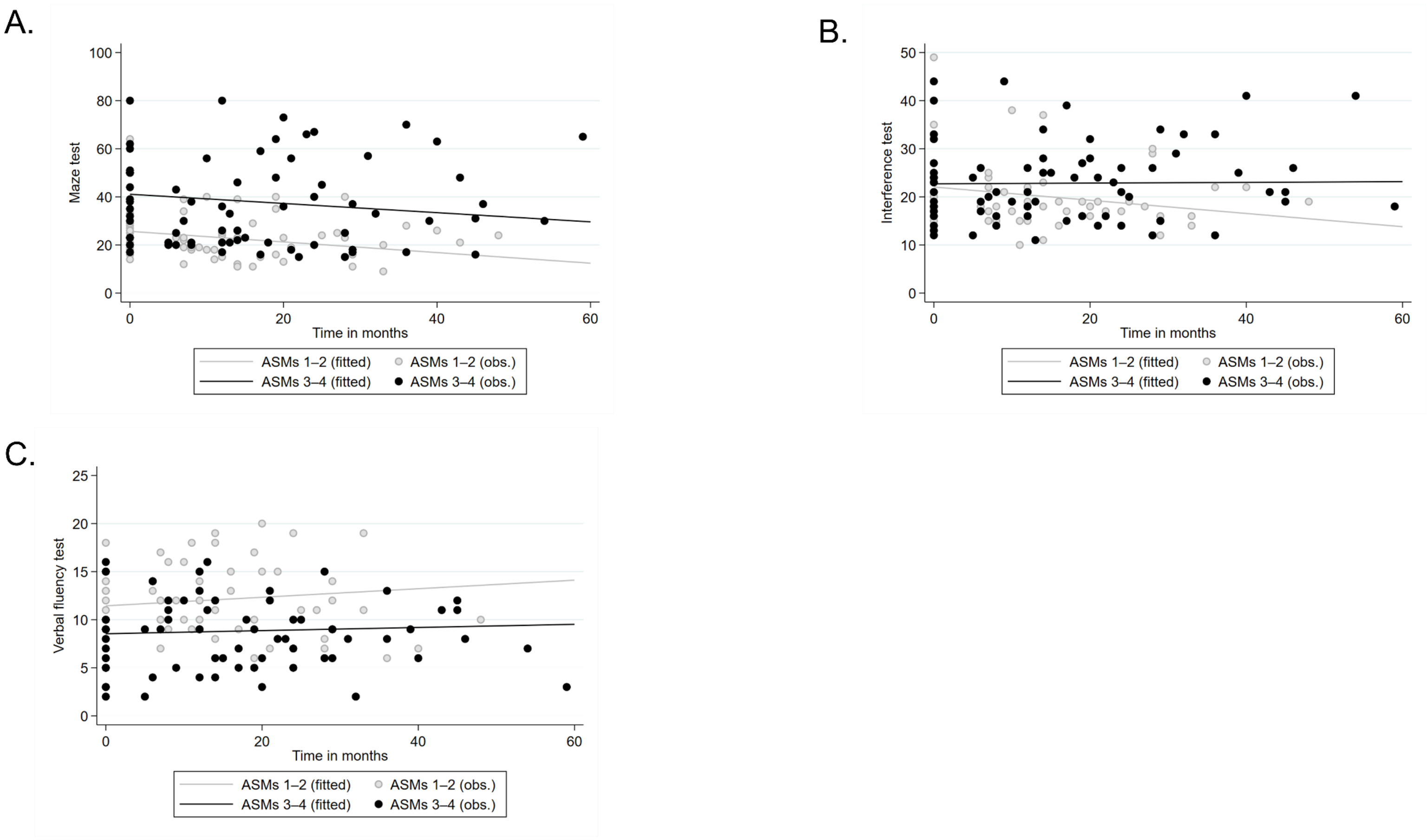

Supplementary Figure 5. Observed and fitted curves for **(A)** maze, **(B)** interference and **(C)** verbal fluency test scores based on linear mixed-effects model over time following VNS therapy based on the number of antiseizure medications in use.
